# 3-(1-Methyl­pyrrolidin-2-yl­idene)-3*H*-indole sesquihydrate

**DOI:** 10.1107/S1600536809047606

**Published:** 2009-11-18

**Authors:** Madeleine Helliwell, Masomeh Aghazadeh, Mehdi M. Baradarani, John A. Joule

**Affiliations:** a The School of Chemistry, The University of Manchester, Manchester M13 9PL, England; b Department of Chemistry, Faculty of Science, University of Urmia, Urmia 57135, Iran

## Abstract

The asymmetric unit of the title compound, C_13_H_14_N_2_·1.5H_2_O, contains two similar mol­ecules of 3-(1-methyl­pyrrolidin-2-yl­idene)-3*H*-indole, (I), and three water mol­ecules. (I) is the product of reacting indole with 1-methyl­pyrrolidin-2-one in the presence of phospho­rus oxychloride. Both organic molecules[Chem scheme1] are almost completely planar; the maximum distances above and below the least-squares plane through all the atoms of mol­ecule 1 are 0.050 (8) and −0.045 (8) Å, respectively, and the deviations for mol­ecule 2 are 0.096 (8) and −0.059 (8) Å, respectively. In the crystal, the two crystallographically different mol­ecules alternate in π-stacked columns [centroid–centroid distances = 3.729 (5) and 3.858 (5) Å], which are linked by O—H⋯N hydrogen bonds to a network of hydrogen-bonded water mol­ecules. O—H⋯O inter­actions are also present.

## Related literature

For the original synthesis of 3-(1-methyl­pyrrolidin-2-yl­idene)-3*H*-indole, see: Youngdale *et al.* (1964 ▶). For a study of its extraordinarily high basicity, see: Harris & Joule (1978 ▶) and for examples of its synthetic applications, see: Bishop *et al.* (1981 ▶, 1982 ▶); Al-Khawaja *et al.* (1984 ▶).
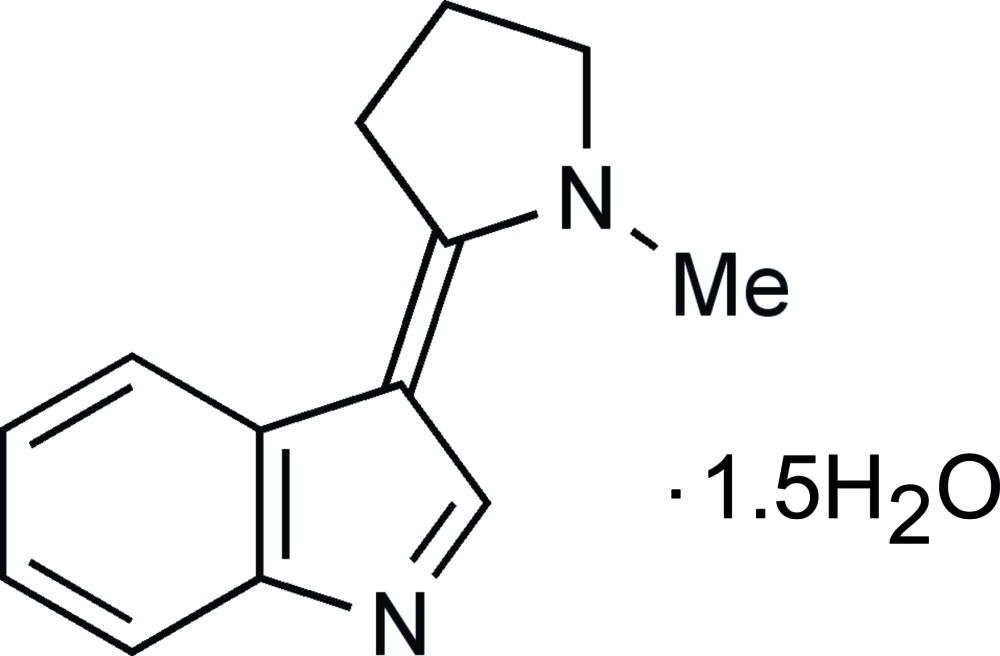


## Experimental

### 

#### Crystal data

C_13_H_14_N_2_·1.5H_2_O
*M* *_r_* = 225.29
Triclinic, 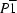

*a* = 7.139 (5) Å
*b* = 10.805 (8) Å
*c* = 15.737 (11) Å
α = 88.460 (13)°
β = 88.163 (14)°
γ = 71.506 (13)°
*V* = 1150.4 (15) Å^3^
*Z* = 4
Mo *K*α radiation
μ = 0.09 mm^−1^
*T* = 100 K
0.60 × 0.06 × 0.06 mm

#### Data collection

Bruker SMART CCD area-detector diffractometer
Absorption correction: none
4171 measured reflections
1814 independent reflections
1183 reflections with *I* > 2σ(*I*)
*R*_int_ = 0.114
θ_max_ = 18.8°

#### Refinement

*R*[*F* ^2^ > 2σ(*F* ^2^)] = 0.078
*wR*(*F* ^2^) = 0.154
*S* = 1.10
1814 reflections
318 parameters
304 restraints
H atoms treated by a mixture of independent and constrained refinement
Δρ_max_ = 0.22 e Å^−3^
Δρ_min_ = −0.21 e Å^−3^

### 

Data collection: *SMART* (Bruker, 2001 ▶); cell refinement: *SAINT* (Bruker, 2002 ▶); data reduction: *SAINT*; program(s) used to solve structure: *SHELXS97* (Sheldrick, 2008 ▶); program(s) used to refine structure: *SHELXL97* (Sheldrick, 2008 ▶); molecular graphics: *SHELXTL* (Sheldrick, 2008 ▶); software used to prepare material for publication: *SHELXTL*.

## Supplementary Material

Crystal structure: contains datablocks I, global. DOI: 10.1107/S1600536809047606/sj2666sup1.cif

Structure factors: contains datablocks I. DOI: 10.1107/S1600536809047606/sj2666Isup2.hkl

Additional supplementary materials:  crystallographic information; 3D view; checkCIF report

## Figures and Tables

**Table 1:** Hydrogen-bond geometry (Å, °)

*D*—H⋯*A*	*D*—H	H⋯*A*	*D*⋯*A*	*D*—H⋯*A*
O2*S*—H4*S*⋯N1	0.91 (3)	1.88 (3)	2.781 (7)	170 (7)
O3*S*—H6*S*⋯N3^i^	0.92 (3)	1.87 (4)	2.733 (7)	156 (6)
O3*S*—H5*S*⋯O2*S*	0.91 (3)	1.86 (3)	2.757 (7)	168 (6)
O2*S*—H3*S*⋯O1*S* ^ii^	0.90 (3)	1.94 (3)	2.807 (7)	160 (6)
O1*S*—H2*S*⋯O3*S* ^iii^	0.91 (3)	1.95 (4)	2.840 (7)	166 (7)
O1*S*—H1*S*⋯O3*S* ^iv^	0.91 (3)	1.90 (4)	2.782 (6)	162 (6)

Symmetry codes: (i) 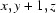; (ii) 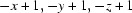; (iii) 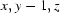; (iv) 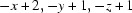.

**Fig. 1.**
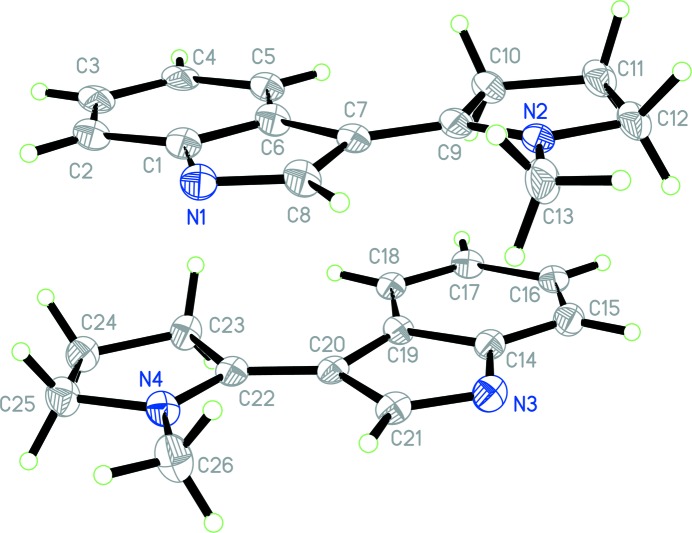
Plot showing the two crystallographically independent molecules of (1), with 50% probability ellipsoids.

**Fig. 2.**
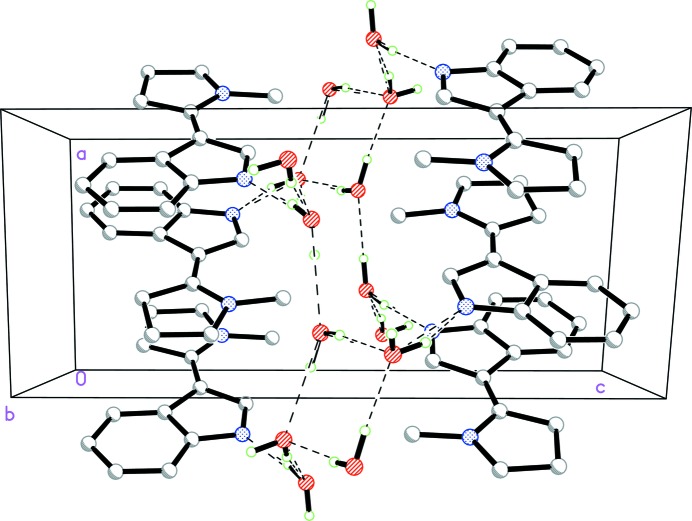
Packing diagram of (1) viewed approximately down b, showing the columns of π-stacked molecules linked by a network of hydrogen bonded water molecules. Only H atoms bonded to water have been included

**Fig. 3.**
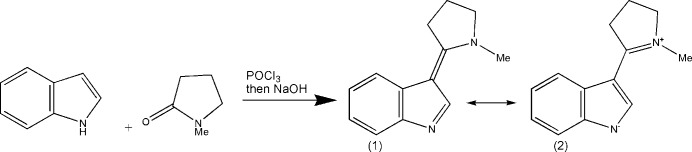
The synthesis of 3-(1-methylpyrrolidin-2-ylidene)-3H-indole

## supplementary crystallographic information

### Comment

Vilsmeier reactions of indole using a tertiary amide in combination with phosphorus oxychloride give rise to 3-acylindoles corresponding to the acyl residue of the amide. However, when 1-methylpyrrolidin-2-one is used as the amide component, 3-(1-methylpyrrolidin-2-ylidene)- 3*H*-indole (1) is the product (Youngdale *et al.*, 1964). We have discussed this structure and studied its strong basicity (Harris & Joule, 1978) and other reactions (Bishop *et al.*, 1981. Bishop *et al.*, 1982. Al-Khawaja *et al.*, 1984). In order to further understand the chemical reactivity of (1) we felt it was important to crystallographically establish the planar structure, predicted by resonance contributor (2), Fig 3, and also to verify the geometry of the exocyclic double bond.

The asymmetric unit of (1) contains two similar molecules of (1), together with three water molecules. The two molecules are essentially planar; the maximum distances above and below the least squares plane through all the atoms of molecule 1 are 0.050 (8) and -0.045 (8) Å for atoms C12 and C11, respectively; the maximum distances above and below the least squares plane through all the atoms of molecule 2 are 0.096 (8) and -0.059 (8) Å, for atoms C24 and C18, respectively. The two ring systems linked by a double bond in each molecule of (1), are essentially coplanar, with dihedral angles of 0.7 (2) between the atoms C1-C8/N1 and C9-C12/N2 and 2.7 (2)° between the atoms C14-C21/N3 and C22-C25/N4. The conjugation between the two nitrogen atoms, as illustrated by (2) is reflected in the bond lengths: in particular, the C8—N1 double bond at 1.311 (8) Å is long for a double bond between carbon and nitrogen, and the C9—N2 single bond at 1.316 (8) Å is correspondingly short, and almost identical in length to that of the nitrogen-carbon double bond; for molecule 2, the respective corresponding distances are 1.320 (8) and 1.322 (8) Å, for C21—N3 and C22—N4. One intriguing aspect of the crystal packing is shown in Figure 2. The two crystallographically different molecules lie one above the other and are parallel to one another, with a dihedral angle between the molecules of 0.6 (1) °. The molecules stack in such a way as to locate the positively charged end of the resonating system (*cf.* (2)) over the negatively charged end of the system in the second molecule. Each molecule forms π-stacking interactions with the crystallographically different molecule above and below it, to form columns along a (Figure 2); the perpendicular distance between the ring N2/C9—C12 in molecule 1 to the ring C14—C19 in molecule 2 is 3.404 Å, with a centroid to centroid distance of 3.729 (5) Å (symmetry equivalent *x*, *y*, *z*), whilst the perpendicular distance of the N2/C9—C12 ring in molecule 1 to the C14—C19 in molecule 2 on the other side of molecule 1 is 3.461 Å, with a centroid to centroid distance of 3.858 (5) Å (symmetry equivalent 1 + *x*, *y*, *z*). Between the π-stack columns of molecules 1 and 2 is a network of H-bonded water molecules, which link the columns together *via* hydrogen bonds between O2S and O3S, and N1 and N3, respectively (see Table 1).

### Experimental

To 1-methyl-2-pyrrolidinone (4 mL, 0.04 mol) cooled in an ice bath was added phosphorous oxychloride (4.08 g, 0.026 mol) with stirring during 30 min. The temperature did not exceed 288 K. The mixture was stirred for an additional 10 min, and then a solution of indole (2.80 g, 0.024 mol) in 1-methyl-2-pyrrolidinone (4 mL) was added slowly during 1 h. The temperature rose to 318 K and a solid separated. The mixture was heated at 353 K for 3 h, and then mixed with water (100 mL). The clear solution was made basic by the addition of sodium hydroxide (6 g) in water (30 mL) causing a solid to separate. The solid was filtered off and washed with water. Recrystallization from ethanol-water afforded 3-(1-methylpyrrolidin-2-ylidene)-3*H*-indole (4.21 g, 90 percent), m.p. 491-492 K.

### Refinement

H atoms bonded to the C atoms were fixed geometrically and treated as riding with C—H = 0.95Å (aromatic), 0.98Å (methyl) and 0.99Å (methylene), with Uiso(H) = 1.2 times those of the parent atoms for the aromatic and methylene H atoms and Uiso(H) = 1.5 times those of the parent atoms for the methyl H atoms. Restraints were applied to the geometry of the water molecules and to the anisotropic thermal parameters of the non-H atoms. The crystal diffracted very weakly, so the data were cut at 1.1 Å resolution.

### Crystal data

**Table:**

C_13_H_14_N_2_·1.5H_2_O	*Z* = 4
*M**_r_* = 225.29	*F*(000) = 484
Triclinic, *P*1	*D*_x_ = 1.301 Mg m^−^^3^
Hall symbol: -P 1	Mo *K*α radiation, λ = 0.71073 Å
*a* = 7.139 (5) Å	Cell parameters from 443 reflections
*b* = 10.805 (8) Å	θ = 2.4–24.5°
*c* = 15.737 (11) Å	µ = 0.09 mm^−^^1^
α = 88.460 (13)°	*T* = 100 K
β = 88.163 (14)°	Needle, colourless
γ = 71.506 (13)°	0.60 × 0.06 × 0.06 mm
*V* = 1150.4 (15) Å^3^	

### Data collection

**Table:**

Bruker SMART CCD area-detector diffractometer	1183 reflections with *I* > 2σ(*I*)
Radiation source: fine-focus sealed tube	*R*_int_ = 0.114
graphite	θ_max_ = 18.8°, θ_min_ = 1.3°
φ and ω scans	*h* = −6→6
4171 measured reflections	*k* = −9→9
1814 independent reflections	*l* = −14→14

### Refinement

**Table:**

Refinement on *F*^2^	Primary atom site location: structure-invariant direct methods
Least-squares matrix: full	Secondary atom site location: difference Fourier map
*R*[*F*^2^ > 2σ(*F*^2^)] = 0.078	Hydrogen site location: inferred from neighbouring sites
*wR*(*F*^2^) = 0.154	H atoms treated by a mixture of independent and constrained refinement
*S* = 1.10	*w* = 1/[σ^2^(*F*_o_^2^) + (0.0362*P*)^2^] where *P* = (*F*_o_^2^ + 2*F*_c_^2^)/3
1814 reflections	(Δ/σ)_max_ = 0.001
318 parameters	Δρ_max_ = 0.22 e Å^−^^3^
304 restraints	Δρ_min_ = −0.21 e Å^−^^3^

### Special details

**Table:**

Geometry. All e.s.d.'s (except the e.s.d. in the dihedral angle between two l.s. planes) are estimated using the full covariance matrix. The cell e.s.d.'s are taken into account individually in the estimation of e.s.d.'s in distances, angles and torsion angles; correlations between e.s.d.'s in cell parameters are only used when they are defined by crystal symmetry. An approximate (isotropic) treatment of cell e.s.d.'s is used for estimating e.s.d.'s involving l.s. planes.
Refinement. Refinement of *F*^2^ against ALL reflections. The weighted *R*-factor *wR* and goodness of fit *S* are based on *F*^2^, conventional *R*-factors *R* are based on *F*, with *F* set to zero for negative *F*^2^. The threshold expression of *F*^2^ > σ(*F*^2^) is used only for calculating *R*-factors(gt) *etc*. and is not relevant to the choice of reflections for refinement. *R*-factors based on *F*^2^ are statistically about twice as large as those based on *F*, and *R*- factors based on ALL data will be even larger.

### Fractional atomic coordinates and isotropic or equivalent isotropic displacement parameters (Å^2^)

**Table:**

	*x*	*y*	*z*	*U*_iso_*/*U*_eq_	
N1	0.7963 (8)	0.5559 (6)	0.3397 (3)	0.0218 (13)	
N2	1.1666 (8)	0.1553 (5)	0.2862 (4)	0.0197 (12)	
C1	0.7801 (10)	0.5929 (7)	0.2549 (5)	0.0210 (13)	
C2	0.6760 (10)	0.7167 (7)	0.2242 (4)	0.0232 (15)	
H2	0.6089	0.7853	0.2614	0.028*	
C3	0.6748 (10)	0.7354 (7)	0.1365 (4)	0.0246 (15)	
H3	0.6085	0.8192	0.1131	0.030*	
C4	0.7688 (10)	0.6334 (7)	0.0826 (5)	0.0237 (15)	
H4	0.7643	0.6481	0.0228	0.028*	
C5	0.8696 (10)	0.5100 (7)	0.1151 (4)	0.0218 (15)	
H5	0.9336	0.4409	0.0777	0.026*	
C6	0.8759 (10)	0.4885 (7)	0.2022 (4)	0.0214 (13)	
C7	0.9652 (10)	0.3799 (7)	0.2581 (4)	0.0182 (13)	
C8	0.9041 (10)	0.4325 (7)	0.3407 (5)	0.0218 (14)	
H8	0.9387	0.3824	0.3917	0.026*	
C9	1.0810 (10)	0.2546 (7)	0.2355 (4)	0.0208 (14)	
C10	1.1279 (10)	0.2148 (6)	0.1464 (4)	0.0238 (15)	
H10A	1.1960	0.2709	0.1167	0.029*	
H10B	1.0057	0.2217	0.1160	0.029*	
C11	1.2626 (10)	0.0733 (7)	0.1498 (4)	0.0257 (15)	
H11A	1.2081	0.0170	0.1163	0.031*	
H11B	1.3965	0.0662	0.1271	0.031*	
C12	1.2691 (10)	0.0343 (7)	0.2431 (4)	0.0247 (15)	
H12A	1.2010	−0.0315	0.2542	0.030*	
H12B	1.4072	−0.0018	0.2619	0.030*	
C13	1.1556 (11)	0.1523 (7)	0.3784 (4)	0.0285 (19)	
H13A	1.1991	0.2225	0.4004	0.043*	
H13B	1.2413	0.0679	0.3996	0.043*	
H13C	1.0190	0.1646	0.3975	0.043*	
N3	0.6780 (8)	0.1334 (5)	0.3039 (3)	0.0217 (13)	
N4	0.3174 (8)	0.5470 (5)	0.3151 (3)	0.0203 (13)	
C14	0.6954 (10)	0.1348 (7)	0.2164 (4)	0.0194 (13)	
C15	0.7912 (10)	0.0279 (7)	0.1667 (5)	0.0233 (15)	
H15	0.8549	−0.0551	0.1918	0.028*	
C16	0.7910 (10)	0.0458 (7)	0.0809 (4)	0.0205 (15)	
H16	0.8565	−0.0254	0.0453	0.025*	
C17	0.6952 (10)	0.1682 (7)	0.0447 (5)	0.0210 (15)	
H17	0.6958	0.1783	−0.0154	0.025*	
C18	0.5994 (9)	0.2753 (7)	0.0939 (4)	0.0179 (14)	
H18	0.5394	0.3586	0.0683	0.022*	
C19	0.5934 (10)	0.2576 (7)	0.1811 (4)	0.0182 (13)	
C20	0.5096 (10)	0.3380 (7)	0.2535 (4)	0.0172 (13)	
C21	0.5704 (10)	0.2530 (7)	0.3247 (4)	0.0188 (14)	
H21	0.5370	0.2794	0.3817	0.023*	
C22	0.3950 (10)	0.4706 (7)	0.2503 (4)	0.0178 (13)	
C23	0.3398 (10)	0.5486 (6)	0.1704 (4)	0.0218 (15)	
H23A	0.2712	0.5061	0.1326	0.026*	
H23B	0.4594	0.5561	0.1400	0.026*	
C24	0.2046 (10)	0.6821 (7)	0.1952 (4)	0.0251 (15)	
H24A	0.0690	0.6961	0.1754	0.030*	
H24B	0.2536	0.7514	0.1703	0.030*	
C25	0.2075 (11)	0.6824 (7)	0.2915 (4)	0.0261 (15)	
H25A	0.2751	0.7431	0.3112	0.031*	
H25B	0.0716	0.7082	0.3163	0.031*	
C26	0.3396 (11)	0.5135 (7)	0.4050 (4)	0.0300 (19)	
H26A	0.2749	0.4479	0.4195	0.045*	
H26B	0.2785	0.5918	0.4386	0.045*	
H26C	0.4803	0.4782	0.4177	0.045*	
O1S	0.7757 (7)	0.1501 (5)	0.5280 (3)	0.0306 (14)	
H1S	0.902 (5)	0.140 (6)	0.543 (4)	0.046*	
H2S	0.779 (9)	0.078 (5)	0.498 (4)	0.046*	
O2S	0.6375 (7)	0.7582 (5)	0.4528 (3)	0.0291 (14)	
H3S	0.508 (5)	0.768 (6)	0.459 (4)	0.044*	
H4S	0.695 (8)	0.686 (5)	0.421 (4)	0.044*	
O3S	0.8359 (7)	0.9358 (5)	0.4183 (3)	0.0286 (14)	
H5S	0.762 (9)	0.881 (6)	0.423 (4)	0.043*	
H6S	0.798 (9)	0.984 (6)	0.369 (3)	0.043*	

### Atomic displacement parameters (Å^2^)

**Table:**

	*U*^11^	*U*^22^	*U*^33^	*U*^12^	*U*^13^	*U*^23^
N1	0.016 (3)	0.023 (3)	0.027 (2)	−0.007 (2)	−0.006 (2)	−0.002 (2)
N2	0.012 (3)	0.020 (3)	0.028 (2)	−0.006 (2)	−0.004 (2)	0.001 (2)
C1	0.012 (3)	0.024 (3)	0.029 (2)	−0.008 (2)	−0.009 (3)	0.000 (2)
C2	0.010 (3)	0.027 (3)	0.033 (3)	−0.005 (3)	−0.009 (3)	0.000 (3)
C3	0.010 (3)	0.029 (3)	0.035 (3)	−0.007 (3)	−0.006 (3)	0.003 (3)
C4	0.010 (3)	0.032 (3)	0.032 (3)	−0.009 (3)	−0.007 (3)	0.003 (2)
C5	0.012 (3)	0.026 (3)	0.028 (3)	−0.007 (3)	−0.008 (3)	0.001 (2)
C6	0.012 (3)	0.025 (2)	0.027 (2)	−0.005 (2)	−0.011 (3)	0.001 (2)
C7	0.010 (3)	0.022 (2)	0.024 (3)	−0.007 (2)	−0.009 (2)	0.001 (2)
C8	0.014 (3)	0.025 (3)	0.026 (3)	−0.005 (3)	−0.010 (3)	0.001 (2)
C9	0.013 (3)	0.022 (3)	0.027 (3)	−0.006 (2)	−0.005 (2)	0.003 (2)
C10	0.016 (3)	0.025 (3)	0.029 (3)	−0.005 (3)	−0.003 (3)	0.001 (2)
C11	0.018 (3)	0.026 (3)	0.032 (3)	−0.004 (3)	−0.007 (3)	−0.003 (3)
C12	0.015 (3)	0.024 (3)	0.034 (3)	−0.003 (3)	−0.010 (3)	−0.003 (2)
C13	0.026 (4)	0.023 (4)	0.029 (3)	0.001 (4)	−0.002 (4)	0.010 (3)
N3	0.020 (3)	0.023 (3)	0.022 (2)	−0.007 (2)	−0.010 (3)	0.005 (2)
N4	0.015 (3)	0.023 (3)	0.022 (2)	−0.004 (2)	−0.007 (2)	0.001 (2)
C14	0.015 (3)	0.021 (3)	0.023 (2)	−0.007 (2)	−0.006 (3)	0.003 (2)
C15	0.020 (3)	0.021 (3)	0.029 (3)	−0.007 (3)	−0.005 (3)	0.002 (2)
C16	0.014 (3)	0.022 (3)	0.030 (3)	−0.010 (3)	−0.005 (3)	−0.003 (3)
C17	0.013 (3)	0.026 (3)	0.028 (3)	−0.010 (3)	−0.011 (3)	−0.002 (2)
C18	0.012 (3)	0.021 (3)	0.024 (3)	−0.008 (3)	−0.009 (3)	0.003 (2)
C19	0.011 (3)	0.021 (3)	0.022 (2)	−0.005 (2)	−0.008 (3)	0.004 (2)
C20	0.013 (3)	0.020 (2)	0.021 (2)	−0.009 (2)	−0.007 (2)	0.003 (2)
C21	0.017 (3)	0.023 (3)	0.019 (3)	−0.010 (3)	−0.008 (3)	0.002 (2)
C22	0.014 (3)	0.022 (2)	0.020 (2)	−0.008 (2)	−0.007 (2)	0.000 (2)
C23	0.019 (3)	0.021 (3)	0.026 (3)	−0.005 (3)	−0.012 (3)	0.003 (2)
C24	0.020 (3)	0.023 (3)	0.031 (3)	−0.004 (3)	−0.008 (3)	0.005 (3)
C25	0.021 (3)	0.024 (3)	0.030 (3)	−0.002 (3)	−0.007 (3)	0.002 (2)
C26	0.035 (5)	0.029 (4)	0.019 (3)	0.001 (4)	0.000 (4)	0.000 (3)
O1S	0.014 (3)	0.036 (4)	0.041 (4)	−0.006 (3)	−0.009 (3)	−0.008 (3)
O2S	0.020 (3)	0.034 (4)	0.035 (4)	−0.009 (3)	−0.005 (3)	−0.004 (3)
O3S	0.021 (3)	0.032 (4)	0.034 (3)	−0.009 (3)	−0.015 (3)	0.011 (3)

### Geometric parameters (Å, °)

**Table:**

N1—C8	1.311 (8)	N4—C26	1.453 (8)
N1—C1	1.381 (8)	N4—C25	1.468 (8)
N2—C9	1.316 (8)	C14—C15	1.388 (9)
N2—C13	1.450 (8)	C14—C19	1.405 (9)
N2—C12	1.454 (8)	C15—C16	1.358 (9)
C1—C2	1.390 (9)	C15—H15	0.9500
C1—C6	1.397 (9)	C16—C17	1.397 (9)
C2—C3	1.389 (9)	C16—H16	0.9500
C2—H2	0.9500	C17—C18	1.384 (9)
C3—C4	1.389 (9)	C17—H17	0.9500
C3—H3	0.9500	C18—C19	1.382 (9)
C4—C5	1.391 (9)	C18—H18	0.9500
C4—H4	0.9500	C19—C20	1.446 (9)
C5—C6	1.384 (9)	C20—C22	1.407 (9)
C5—H5	0.9500	C20—C21	1.418 (9)
C6—C7	1.436 (9)	C21—H21	0.9500
C7—C9	1.393 (9)	C22—C23	1.486 (9)
C7—C8	1.431 (9)	C23—C24	1.512 (9)
C8—H8	0.9500	C23—H23A	0.9900
C9—C10	1.475 (9)	C23—H23B	0.9900
C10—C11	1.527 (9)	C24—C25	1.518 (9)
C10—H10A	0.9900	C24—H24A	0.9900
C10—H10B	0.9900	C24—H24B	0.9900
C11—C12	1.514 (9)	C25—H25A	0.9900
C11—H11A	0.9900	C25—H25B	0.9900
C11—H11B	0.9900	C26—H26A	0.9800
C12—H12A	0.9900	C26—H26B	0.9800
C12—H12B	0.9900	C26—H26C	0.9800
C13—H13A	0.9800	O1S—H1S	0.91 (3)
C13—H13B	0.9800	O1S—H2S	0.91 (3)
C13—H13C	0.9800	O2S—H3S	0.90 (3)
N3—C21	1.320 (8)	O2S—H4S	0.91 (3)
N3—C14	1.379 (8)	O3S—H5S	0.91 (3)
N4—C22	1.322 (8)	O3S—H6S	0.92 (3)
			
C8—N1—C1	105.4 (6)	C22—N4—C25	114.9 (5)
C9—N2—C13	127.0 (6)	C26—N4—C25	117.8 (5)
C9—N2—C12	114.8 (6)	N3—C14—C15	125.5 (7)
C13—N2—C12	118.0 (6)	N3—C14—C19	111.9 (6)
N1—C1—C2	125.1 (7)	C15—C14—C19	122.4 (7)
N1—C1—C6	111.6 (6)	C16—C15—C14	117.9 (7)
C2—C1—C6	123.3 (7)	C16—C15—H15	121.0
C3—C2—C1	116.9 (7)	C14—C15—H15	121.0
C3—C2—H2	121.6	C15—C16—C17	120.4 (7)
C1—C2—H2	121.6	C15—C16—H16	119.8
C2—C3—C4	121.1 (7)	C17—C16—H16	119.8
C2—C3—H3	119.5	C18—C17—C16	122.0 (7)
C4—C3—H3	119.5	C18—C17—H17	119.0
C3—C4—C5	120.8 (7)	C16—C17—H17	119.0
C3—C4—H4	119.6	C19—C18—C17	118.2 (7)
C5—C4—H4	119.6	C19—C18—H18	120.9
C6—C5—C4	119.6 (7)	C17—C18—H18	120.9
C6—C5—H5	120.2	C18—C19—C14	118.9 (6)
C4—C5—H5	120.2	C18—C19—C20	136.4 (7)
C5—C6—C1	118.3 (7)	C14—C19—C20	104.7 (6)
C5—C6—C7	135.7 (7)	C22—C20—C21	129.8 (6)
C1—C6—C7	105.9 (6)	C22—C20—C19	125.9 (6)
C9—C7—C8	129.5 (6)	C21—C20—C19	104.3 (6)
C9—C7—C6	127.5 (6)	N3—C21—C20	113.4 (6)
C8—C7—C6	103.0 (6)	N3—C21—H21	123.3
N1—C8—C7	114.1 (6)	C20—C21—H21	123.3
N1—C8—H8	123.0	N4—C22—C20	127.5 (6)
C7—C8—H8	123.0	N4—C22—C23	108.2 (6)
N2—C9—C7	127.9 (6)	C20—C22—C23	124.3 (6)
N2—C9—C10	109.2 (6)	C22—C23—C24	107.1 (6)
C7—C9—C10	122.9 (6)	C22—C23—H23A	110.3
C9—C10—C11	106.1 (5)	C24—C23—H23A	110.3
C9—C10—H10A	110.5	C22—C23—H23B	110.3
C11—C10—H10A	110.5	C24—C23—H23B	110.3
C9—C10—H10B	110.5	H23A—C23—H23B	108.5
C11—C10—H10B	110.5	C23—C24—C25	105.0 (5)
H10A—C10—H10B	108.7	C23—C24—H24A	110.7
C12—C11—C10	105.0 (6)	C25—C24—H24A	110.7
C12—C11—H11A	110.7	C23—C24—H24B	110.7
C10—C11—H11A	110.7	C25—C24—H24B	110.7
C12—C11—H11B	110.7	H24A—C24—H24B	108.8
C10—C11—H11B	110.7	N4—C25—C24	104.1 (5)
H11A—C11—H11B	108.8	N4—C25—H25A	110.9
N2—C12—C11	104.2 (6)	C24—C25—H25A	110.9
N2—C12—H12A	110.9	N4—C25—H25B	110.9
C11—C12—H12A	110.9	C24—C25—H25B	110.9
N2—C12—H12B	110.9	H25A—C25—H25B	109.0
C11—C12—H12B	110.9	N4—C26—H26A	109.5
H12A—C12—H12B	108.9	N4—C26—H26B	109.5
N2—C13—H13A	109.5	H26A—C26—H26B	109.5
N2—C13—H13B	109.5	N4—C26—H26C	109.5
H13A—C13—H13B	109.5	H26A—C26—H26C	109.5
N2—C13—H13C	109.5	H26B—C26—H26C	109.5
H13A—C13—H13C	109.5	H1S—O1S—H2S	107 (4)
H13B—C13—H13C	109.5	H3S—O2S—H4S	108 (4)
C21—N3—C14	105.7 (6)	H5S—O3S—H6S	106 (4)
C22—N4—C26	127.3 (6)		
			
C8—N1—C1—C2	179.4 (7)	C21—N3—C14—C15	−177.6 (7)
C8—N1—C1—C6	1.7 (8)	C21—N3—C14—C19	−1.0 (8)
N1—C1—C2—C3	−179.4 (7)	N3—C14—C15—C16	178.7 (6)
C6—C1—C2—C3	−1.9 (11)	C19—C14—C15—C16	2.5 (11)
C1—C2—C3—C4	1.8 (10)	C14—C15—C16—C17	−0.6 (10)
C2—C3—C4—C5	−0.9 (11)	C15—C16—C17—C18	0.7 (11)
C3—C4—C5—C6	0.1 (11)	C16—C17—C18—C19	−2.5 (11)
C4—C5—C6—C1	−0.1 (10)	C17—C18—C19—C14	4.2 (10)
C4—C5—C6—C7	−178.3 (8)	C17—C18—C19—C20	−178.5 (7)
N1—C1—C6—C5	178.9 (6)	N3—C14—C19—C18	179.0 (6)
C2—C1—C6—C5	1.1 (11)	C15—C14—C19—C18	−4.4 (11)
N1—C1—C6—C7	−2.5 (8)	N3—C14—C19—C20	0.9 (8)
C2—C1—C6—C7	179.8 (6)	C15—C14—C19—C20	177.6 (6)
C5—C6—C7—C9	0.0 (14)	C18—C19—C20—C22	1.9 (13)
C1—C6—C7—C9	−178.3 (7)	C14—C19—C20—C22	179.5 (6)
C5—C6—C7—C8	−179.5 (8)	C18—C19—C20—C21	−178.0 (8)
C1—C6—C7—C8	2.1 (7)	C14—C19—C20—C21	−0.4 (7)
C1—N1—C8—C7	−0.2 (8)	C14—N3—C21—C20	0.7 (8)
C9—C7—C8—N1	179.3 (7)	C22—C20—C21—N3	179.9 (7)
C6—C7—C8—N1	−1.2 (8)	C19—C20—C21—N3	−0.2 (8)
C13—N2—C9—C7	1.9 (11)	C26—N4—C22—C20	2.9 (12)
C12—N2—C9—C7	176.7 (7)	C25—N4—C22—C20	178.9 (6)
C13—N2—C9—C10	−178.9 (6)	C26—N4—C22—C23	−177.4 (6)
C12—N2—C9—C10	−4.1 (8)	C25—N4—C22—C23	−1.4 (8)
C8—C7—C9—N2	−2.1 (12)	C21—C20—C22—N4	0.3 (12)
C6—C7—C9—N2	178.5 (7)	C19—C20—C22—N4	−179.5 (7)
C8—C7—C9—C10	178.8 (7)	C21—C20—C22—C23	−179.3 (7)
C6—C7—C9—C10	−0.6 (11)	C19—C20—C22—C23	0.8 (11)
N2—C9—C10—C11	−1.2 (8)	N4—C22—C23—C24	−3.9 (8)
C7—C9—C10—C11	178.0 (7)	C20—C22—C23—C24	175.8 (6)
C9—C10—C11—C12	5.7 (7)	C22—C23—C24—C25	7.3 (8)
C9—N2—C12—C11	7.7 (8)	C22—N4—C25—C24	6.0 (8)
C13—N2—C12—C11	−177.0 (6)	C26—N4—C25—C24	−177.6 (6)
C10—C11—C12—N2	−7.6 (7)	C23—C24—C25—N4	−7.8 (7)

### Hydrogen-bond geometry (Å, °)

**Table:**

*D*—H···*A*	*D*—H	H···*A*	*D*···*A*	*D*—H···*A*
O2S—H4S···N1	0.91 (3)	1.88 (3)	2.781 (7)	170 (7)
O3S—H6S···N3^i^	0.92 (3)	1.87 (4)	2.733 (7)	156 (6)
O3S—H5S···O2S	0.91 (3)	1.86 (3)	2.757 (7)	168 (6)
O2S—H3S···O1S^ii^	0.90 (3)	1.94 (3)	2.807 (7)	160 (6)
O1S—H2S···O3S^iii^	0.91 (3)	1.95 (4)	2.840 (7)	166 (7)
O1S—H1S···O3S^iv^	0.91 (3)	1.90 (4)	2.782 (6)	162 (6)

Symmetry codes: (i) *x*, *y*+1, *z*; (ii) −*x*+1, −*y*+1, −*z*+1; (iii) *x*, *y*−1, *z*; (iv) −*x*+2, −*y*+1, −*z*+1.
